# Differential Cognitive Performance in Females and Males with Regular Cannabis Use

**DOI:** 10.1017/S1355617721000606

**Published:** 2021-07

**Authors:** Ashley M. Schnakenberg Martin, Deepak Cyril D’Souza, Sharlene D. Newman, William P. Hetrick, Brian F. O’Donnell

**Affiliations:** 1 Department of Psychological and Brain Sciences, Indiana University, Bloomington, Bloomington, Indiana, USA; 2 Psychology Service, VA Connecticut Healthcare System, West Haven, Connecticut, USA; 3 Department of Psychiatry, Yale University School of Medicine, New Haven, Connecticut, USA; 4 Psychiatry Service, VA Connecticut Healthcare System, West Haven, Connecticut, USA; 5 Program in Neuroscience, Indiana University, Bloomington, Bloomington, Indiana, USA; 6 Department of Psychiatry, Indiana University School of Medicine, Indianapolis, Indiana, USA

**Keywords:** Cannabis, Cognition, Sex differences, Intelligence, Psychomotor speed, Verbal learning

## Abstract

**Objectives:**

Preclinical and clinical studies suggest that males and females may be differentially affected by cannabis use. This study evaluated the interaction of cannabis use and biological sex on cognition, and the association between observed cognitive deficits and features of cannabis use.

**Methods:**

Cognitive measures were assessed in those with regular, ongoing, cannabis use (*N* = 40; 22 female) and non-using peers (*N* = 40; 23 female). Intelligence, psychomotor speed, and verbal working memory were measured with the Wechsler Abbreviated Scale of Intelligence, Digit Symbol Test, and Digit Span and Hopkins Verbal Learning Test, respectively. Associations between cognitive measures and cannabis use features (e.g., lifetime cannabis use, age of initiation, time since last use of cannabis, recent high-concentration tetrahydrocannabinoid exposure) were also evaluated.

**Results:**

No main effects of group were observed across measures. Significant interactions between group and biological sex were observed on measures of intelligence, psychomotor speed, and verbal learning, with greatest group differences observed between males with and without regular cannabis use. Psychomotor performance was negatively correlated with lifetime cannabis exposure. Female and male cannabis use groups did not differ in features of cannabis use.

**Conclusions:**

Findings suggest that biological sex influences the relationship between cannabis and cognition, with males potentially being more vulnerable to the neurocognitive deficits related to cannabis use.

## INTRODUCTION

Cannabis is the most widely used substance in the world (Johnston, O’Malley, Bachman, & Schulenberg, 2012; SAMHSA, 2011). Largely due to the primary psychoactive component, delta-9-tetrahydrocannabinoid (Δ−9-THC), cannabis generates psychotomimetic effects, cognitive dysfunction, psychomotor deficits, and increased risk for the development of schizophrenia, depression, and anxiety (Radhakrishnan, Wilkinson, & D’Souza, 2014). Understanding the chronic effects of cannabis on cognition is necessary, especially as cannabis use is expected to increase (Carliner, Brown, Sarvet, & Hasin, 2017; Hasin et al., 2015), likely related to a decreasing perception of risk (Azofeifa, 2016; Carliner et al., 2017; Zehra et al., 2018) and liberalization of cannabis laws.

Both acute and chronic cannabis exposure have been associated with poorer neurocognitive performance in executive function, verbal learning and memory, attention, and psychomotor function, and these deficits have also been observed after periods of abstinence (Broyd, van Hell, Beale, Yuecel, & Solowij, 2016; Dahlgren, Sagar, Racine, Dreman, & Gruber, 2016; Gruber & Sagar, 2017; Hanson et al., 2010; Messinis, Kyprianidou, Malefaki, & Papathanasopoulos, 2006; Ranganathan & D’Souza, 2006). Additionally, in a large prospective study, regular cannabis use (compared to a cannabis naïve baseline) was associated with declines in cognitive function, specifically in domains of intelligence, executive function, and processing speed (Meier et al., 2012). It is important to acknowledge that there are mixed findings regarding the relationship between cognition and cannabis. For example, investigations using quasi-experimental twin-study designs have observed minimal relationships between cannabis and declines in cognition (Meier et al., 2018; Ross et al., 2020). Also, a recent meta-analysis (Figueiredo, Tolomeo, Steele, & Baldacchino, 2020) suggested that cannabis use is associated with small effect sizes over a spectrum of cognitive measures, with the largest effect sizes for short-term (*d* = .48) and long-term (*d* = .43) memory deficits. A meta-analysis by Scott et al. (2018) further indicated that the modest cognitive impairments shown by cannabis users are further diminished after abstinence longer than 72 h (Scott et al., 2018).

The mixed literature on the relationship between cognition and cannabis may in part be due to the failure to consider biological sex as a potential moderating variable. While historically the prevalence of cannabis use has been two to three times higher in males than females, in the 21^st^ century, prevalence rates have been converging in the United States (Chapman et al., 2017; Johnson et al., 2015; Nia, Mann, Kaur, & Ranganathan, 2018). Clinical and preclinical data have observed differences in behavioral effects and development of tolerance to cannabis. These differences may be related to sex-dependent differences in cannabinoid metabolism and influence of sex-specific hormones and/or endocannabinoids (Fattore & Fratta, 2010; Fratta & Fattore, 2013; Ketcherside, Baine, & Filbey, 2016; Nia et al., 2018; Paola Castelli et al., 2014; Prashad, Hammonds, Wiese, Milligan, & Filbey, 2020). Further, few studies have systematically investigated sex differences as a moderator of the relationship between cannabis use and observed cognitive deficits. In acute challenge studies with the administration of cannabinoids, some studies failed to find an impact of biological sex on cognitive measures, such as impulsivity, selective and divided attention, cognitive flexibility, or time estimation (Anderson, Rizzo, Block, Pearlson, & O’Leary, 2010; McDonald, Schleifer, Richards, & de Wit, 2003). Others found that women appeared to be more sensitive to the acute effects of cannabinoids on psychomotor function (Roser et al., 2009), while showing enhancement in spatial working memory (Makela et al., 2006).

Similarly, mixed findings have been observed regarding the relationship between sex, chronic cannabis use, and cognition. Chronic cannabis use has been associated with greater deficits in psychomotor and cognitive speed in males, compared to females, after one week of abstinence (Lisdahl & Price, 2012). In contrast, greater visuospatial memory deficits were observed in females (Pope, Jacobs, Mialet, Yurgelun-Todd, & Gruber, 1997), which may be associated with age of initiation of cannabis use (Noorbakhsh, Afzali, Boers, & Conrod, 2020). Additionally, a series of studies by Crane and colleagues demonstrated that cannabis use was more strongly associated with deficits in episodic memory in females compared to males, although more cannabis use was correlated with poor decision-making in males, but not females (Crane, Schuster, & Gonzalez, 2013; Crane, Schuster, Mermelstein, & Gonzalez, 2015). Crane et al. (2015) also observed that in women, but not men, earlier onset of cannabis use was associated with greater memory deficits. Additionally, in women, earlier onset of cannabis use was associated with lower estimated intelligence, although this was observed at the trend (*p* = .10) level (Crane et al., 2015).

Neuroimaging investigations have also demonstrated sex differences on the neurobiological impact of regular cannabis use. For example, increased amygdala volumes and worse depression and anxiety have been observed in female users (McQueeny et al., 2011). Also, group by biological sex interactions has been observed such that female cannabis users had larger prefrontal cortex volumes and males had smaller volumes compared to non-using peers (Medina et al., 2009). Electrophysiological investigations of early sensory processing revealed altered visual steady state evoked potentials in females, but not males, suggesting altered primary visual neural circuits in women with regular cannabis use (Skosnik, Krishnan, Vohs, & O’Donnell, 2006). Finally, previous work from our laboratory using magnetic resonance spectroscopy suggested that glutamate levels may be dependent on recent cannabis use, and further that this relationship may be modulated by sex (Newman et al., 2019).

While each of the studies investigating biological sex as a moderator of the relationship between cognition and cannabis use made unique scientific contributions, there remain gaps in the literature. For example, the literature would benefit from systematic comparison of current, regular cannabis users to non-using peers (a control group). While investigation after periods of abstinence is important, prolonged abstinence may represent a different cognitive state compared to regular, recent use, should any deficits recover with abstinence. The current study sought to address these limitations. This study investigated cognitive function in males and females with regular cannabis (CB) use compared to non-using healthy controls (HC). We hypothesized that i) the group with regular CB use would have decreased cognitive performance, compared to the HC group, especially in domains of memory, ii) cognitive performance would be moderated by sex, and iii) earlier age of onset of cannabis use and total lifetime exposure would be associated with worse cognitive performance.

## METHODS

### Subjects

Forty current cannabis users (CB; 22 female) and forty healthy controls (HC; 23 female) were recruited from the community with local advertisements and via word of mouth.

Subjects were determined to be free of any current Axis I disorder as per the Structured Clinical Interview for Diagnostic and Statistical Manual, 4th. Edition (DSM-IV)-TR (SCID), Research Version (First, Spitzer, Gibbon, & Williams, 2002), except cannabis abuse or dependence. Exclusion criteria for all subjects included lifetime dependence of any substance (excluding nicotine and cannabis). All participants were 18 years of age or older, and free of any neurological disorder, head injury with loss of consciousness greater than five minutes, learning disability, and family history of a first degree relative with psychosis. Those in the CB group had a current rate of cannabis use ≥ 1×/week for a minimum of the past 3 months. Subjects in the healthy control group were required to have no cannabis use in the past 3 months, no history of cannabis DSM-IV abuse or dependence, a negative urine screen, and lifetime total use of <16 exposures. All subjects also underwent urine toxicology to validate self-report regarding current CB use and verify abstinence from other illicit substances.

### Procedures

Participants were recruited via local advertisements and word of mouth, to which they were instructed to contact the laboratory by phone for a brief phone screen to assess general eligibility. Potentially eligible subjects were invited into the laboratory for a full screening evaluation and study participation. All participants were asked to refrain from recent use of alcohol, cannabis, and other illicit substances from the evening prior to their scheduled test day (~12 h before testing). After the informed consent process, subjects underwent a diagnostic clinical interview with the SCID, Research Version (First et al., 2002). Subjects then completed a series of self-report measures assessing lifetime substance use. Eligible subjects were then administered tests of cognition. For details of cognitive assessments, see below. Total study participation took approximately 4–5 h. Subjects were compensated $12/hour for the initial interview, questionnaires, and cognitive testing.

### Measures

#### Substance use

A Substance Use Questionnaire (SUQ), as used in previous studies by our laboratory (Fridberg, Vollmer, O’Donnell, & Skosnik, 2011; Skosnik et al., 2012), was used to collect information about many substances, including tobacco, caffeine, alcohol, cannabis, synthetic marijuana (i.e., spice, K2), sedatives/tranquilizers, ecstasy, speed, cocaine, opiates, hallucinogens, salvia divinorum, ketamine, phencyclidine, inhalants, and gamma-Hydroxybutyric acid. The SUQ also includes a fictitious substance, relevin, which if endorsed was used to exclude subjects for inaccurate over-reporting. None of the subjects in the study endorsed using this sham substance. The SUQ was used to determine the total lifetime exposures of cannabis use (number of occasions in which an individual used cannabis), age of cannabis initiation, number of occasions of uses in the past month (recent use), days since last use of cannabis, if subjects ever used wax, and if they used wax, the number of occasions in which wax was used over the past 6 months. Additionally, subjects were asked to report their last use all substances, including cannabis, alcohol, tobacco, and caffeine.

#### Cognition

Intellectual function was assessed using the full-scale intelligence quotient (IQ) derived from the Vocabulary and Matrix Reasoning subtests from the Wechsler Abbreviated Scale of Intelligence (WASI) (Wechsler, 1987). Psychomotor performance was measured with the total score from the Digit Symbol Test from the Wechsler Adult Intelligence Scale III (WAIS III) (Tulsky, Ivnik, Price, & Wilkins, 2003). Verbal working memory was assessed with the Digit Span from the Wechsler Adult Intelligence Scale III total score measured by the correct number of trials summed between the forward and backward blocks. The Hopkins Verbal Learning Task – Revised (HVLT-R) (Benedict, Schretlen, Groninger, & Brandt, 1998) assessed verbal learning. In the HVLT-R, subjects were read a list of 12 words and then asked to repeat the word list back over a series of 3 trials. The sum of the three trials represented the total immediate recall score. After a 20-minute delay, subjects were asked to recall as many words as they could remember from the list, which was the delayed recall total score. A forced choice selection list was then read to subjects with the instructions to indicate words that were or were not on the list. The total correct number of hits was the cued recognition recall score. The retention percentage t-score was then determined using the scoring appendix, based on the ratio of words retained between the higher score on trials 2 or 3 and the total delayed recall. Finally, a recognition discrimination index t-score was also determined using the scoring appendix, based on the ratio of the number of true positives and false positives identified during cued recognition recall.

### Statistical Analysis

All statistical analyses were performed with SPSS statistical software from IBM (CORP, 2020). The Student’s t-test was used to assess for differences in age, BMI, and education. A chi-square test was used to assess for differences in biological sex, ethnicity, and race between cannabis users and controls (Table 1) and between male and female cannabis users (Table 2).

Two-way analysis of covariance (ANCOVA) was used to assess group differences and interactions between biological sex and cannabis use. Education was used a covariate across analyses. In the presence of significant interaction effects between sex and group, the education-adjusted scores from the ANCOVA were submitted to post-hoc testing with Sidak adjustments to correct for multiple comparisons. Effect sizes of post-hoc group comparisons were measured with Cohen’s *d*. A Cohen’s *d* ≤ .2 was considered small, ≤.4 medium, and ≤.8 large (Cohen, 1988). To limit the number of tests performed, we also conducted one MANCOVA (controlling for education) with the five HVLT outcomes and findings remained consistent with the results presented below. Finally, for cognitive domains in which a significant main or interaction effect was observed on a cognitive measure, exploratory partial correlations controlling for education were used to assess associations between cognitive measures and features of cannabis use, including total lifetime exposure, age of initiation, time since last use, recent (past month) use, lifetime wax (high-concentration THC) exposure, and wax use over the past 6 months.

## RESULTS

### Subjects

As per independent samples t-tests and chi-square testing, groups did not differ in age (CB = 21.1 ± 3.7 years; HC = 22.7 ± 4.4 years), sex (CB = 18 males: 22 females; HC = 17 males: 23 females), race, or BMI (Table 1). Groups did significantly differ in years of completed education (*p* = .002). CB and HC groups did not differ in time since last use of alcohol, tobacco, or caffeine (*p* > .05). CB and HC groups did not differ in number of cigarettes per day (HC = .001 ± .01; CB = .74 ± 2.66). Although groups differed in number of alcoholic drinks over the past week (HC = 2.34 ± 3.78; CB = 5.81 ± 7.65; F(72) = 3.9, *p* = .02), groups did not differ on the Short-Michigan Alcohol Screening Test (SMAST), a measure of problematic drinking (*p* > .05). Controlling for number of drinks in the last week, in addition to education, did not change presented study findings which only controlled for education. More than half of the subjects (23/40) in the CB group met criteria for a DSM-IV lifetime diagnosis of CB abuse or dependence. Data for two participants in the CB group were insufficient to make a definitive diagnostic determination regarding CB abuse or dependence. Male and female CB use groups did not differ in their features of CB use (Table 2). Thirty-six subjects in the CB group had a positive urine toxicology for cannabinoids and zero in the HC group. Notably, groups did not differ in number of cigarettes per day, drinks over the past week, or in problematic drinking based on the SMAST (*p* > .05).

**Fig. 1.**
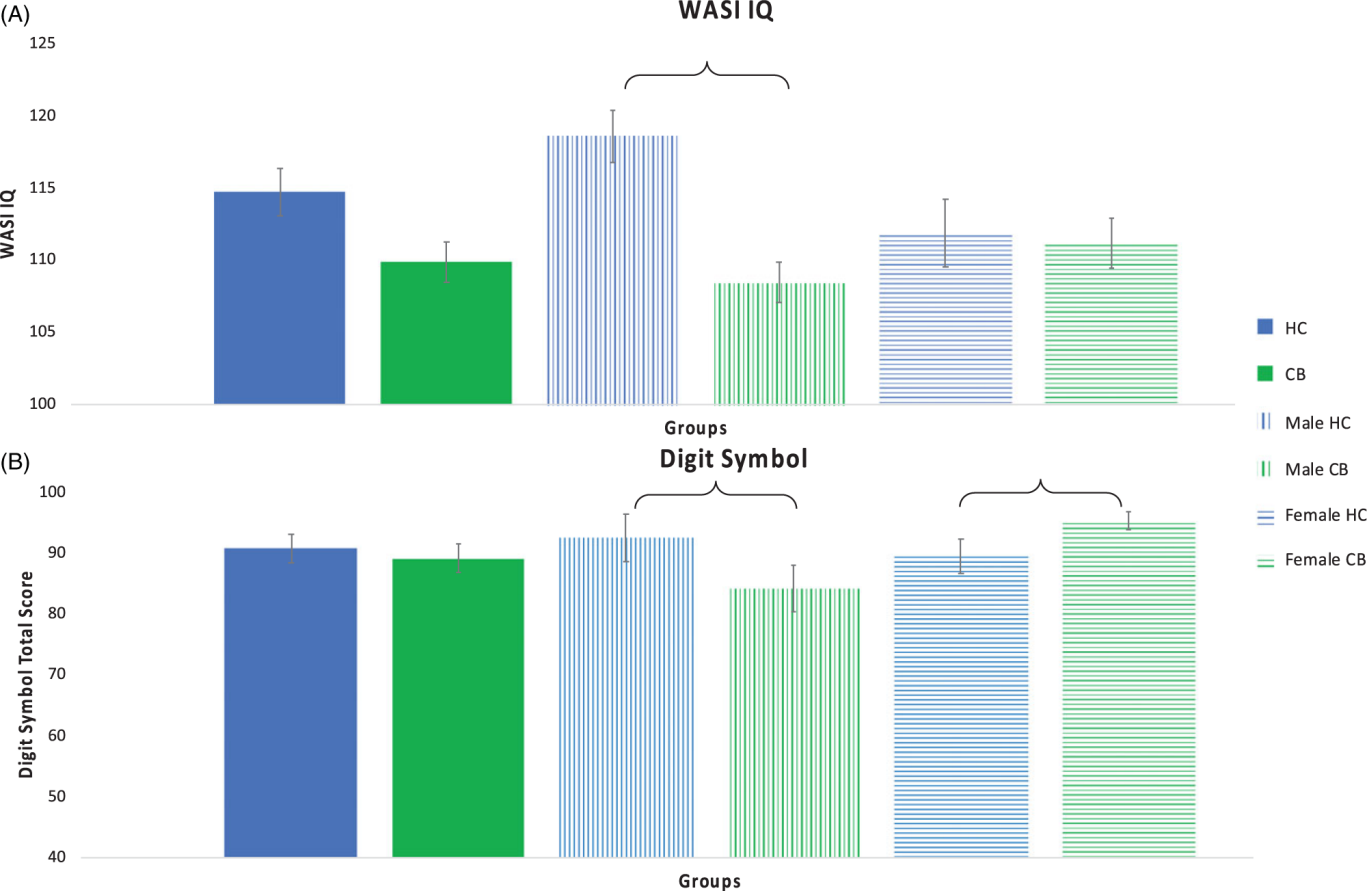
Intellectual and Psychomotor Function. Intellectual function as measured with the WASI IQ (Panel A) and psychomotor function as measured with the Digit Symbol (Panel B). Significant differences between groups are indicated with a bracket. Error bars shown with standard error.

**Fig. 2.**
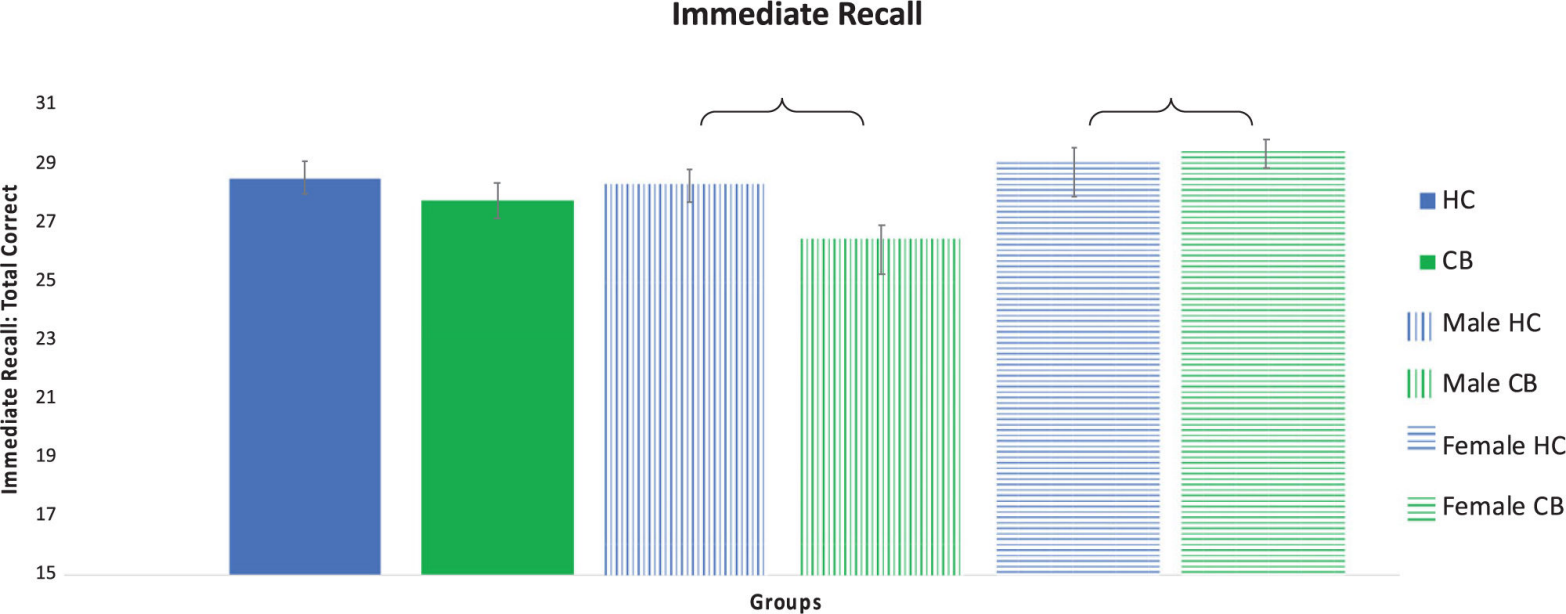
Verbal Learning and Memory. Verbal learning and memory as measured with the Hopkins Verbal Learning Test (HVLT) total immediate recall. Significant differences between groups indicated with a bracket. Error bars shown with standard error.

Significant differences were observed between the cannabis and control groups by sex on measures of intellectual function, psychomotor function, and verbal learning and memory (Table 3; Figures 1 and 2).

#### WASI IQ:

There was trend for a main effect of group on WASI IQ (*F*(1, 75) = 3.642, *p* = .060) but not for biological sex (*F*(1,75) = 1.078, *p* = .302). There was a significant interaction between group and biological sex (*F*(1,75) = 5.303, *p* = .024), such that the male CB group performed worse than male HCs. Post-hoc testing revealed that the male CB group was observed to have significantly lower scores compared to the male HC group (*p* < .001). There was no statistically significant difference between the two female groups (*p* = .797). Measures of effect sizes between the HC and CB groups, male groups, and female groups were Cohen’s *d* = .50, 1.18, and .069, respectively.

#### Digit Symbol:

Main effects for group (Cohen’s *d* = .11) and sex were not significant (*p* > .05). There was a significant interaction between group and biological sex (*F*(1,75) = 5.204, *p* = .009), such that the male CB group performed worse than male HCs and the female HCs performed worse than the female CB group. Post-hoc testing revealed that the male CB group had significantly lower scores compared to the male HC group (*p* < .001; Cohen’s *d* = .68). Additionally, the female CB group was observed to have significantly higher scores compared to the female HC group (*p* < .001; Cohen’s *d* = .50).

#### Digit Span:

Main effect of group was not significant (*p* > .05). There was a significant main effect of biological sex (*F*(1,75) = 4.766, *p* = .032), indicating that females had higher scores than males. The interaction between group and sex was not significant (*p* > .05). Measures of effect sizes between the HC and CB groups, male groups, and female groups were Cohen’s *d* = .14, .50, and .08, respectively.

#### HVLT: Total Recall: immediate and delayed:

There were no main effects of group on total immediate recall (HC vs CB Cohen’s *d* = .21) or total delayed free recall (HC vs CB Cohen’s *d* = .41). There was a main effect of biological sex for total immediate recall (*F*(1,75) = 5.941, *p* = .017), but not for total delayed free recall (*F*(1,75) = 2.255, *p* = .137). A significant interaction between group and biological sex was observed for total immediate recall (*F*(1,75) = 3.955, *p* = .050) and total delayed free recall (*F*(1,75) = 7.006, *p* = .010). Post-hoc testing for total immediate recall demonstrated that the male CB group recalled significantly fewer words than the male HC group (*p* < .001; Cohen’s *d* = .77), whereas female CB users performed better than female controls (*p* = .02; Cohen’s *d* = .17). Post-hoc testing for delayed free recall demonstrated that the male CB group recalled significantly fewer correct responses compared to the male HC group (*p* < .001; Cohen’s *d* = 1.03), while the two female groups did not differ (*p* > .05; Cohen’s *d* = .08).

#### HVLT: Cued Recognition Recall: “hits” forced choice:

The main effect of group was not significant (HC vs CB Cohen’s *d* = .07). There was a main effect of biological sex (*F*(1,75) = 5.839, *p* = .018), indicating that females had greater cued recognition recall than males. The interaction between group and biological sex was also not significant (*p* > .05). Measures of effect sizes between male groups and female groups were Cohen’s *d* = .27 and .27, respectively.

#### HVLT: Retention % [(delayed recall (trial 4)/higher score of trials 2 or 3) × 100] T-Score:

Main effects of group (HC vs CB Cohen’s *d* = .32) and biological sex were not significant (*p* > .05). A significant interaction between group and biological sex (*F*(1,75) = 4.523, *p* = .037) was observed. Post-hoc testing revealed that the male CB group showed lower retention compared to the male HC group (*p* < .001; Cohen’s *d* = .79), while the two female groups did not differ (*p* > .05; Cohen’s *d* = .11).

#### HVLT: Recognition Discrimination Index (Total no of true positives-total no of false-positives):

The main effect of group was not significant (HC vs CB Cohen’s *d* = .24). A significant main effect was observed for biological sex (*F*(1,75) = 9.854, *p* = .002). The interaction between group and sex was not statistically significant (*p* > .05). Measures of effect sizes between the male groups and female groups were Cohen’s *d* = .50, and .11, respectively. Notably, groups were not significantly different in their recognition discrimination index (RD) scores (*p* > .05). Only one participant (CB group) had an *RD* = 7, two (one in each of the CB and HC groups) had a score of 9, while all others were 10+. Thus, these RD scores were considered as indication of credible responding by study participants (Abeare et al., 2020; Bailey, Soble, Bain, & Fullen, 2018; Sawyer, Testa, & Dux, 2017).

#### Relationship between observed cognitive performance and features of cannabis use:

For all cannabis use group participants (male CB + female CB), the only observed significant correlation was a negative association between digit symbol and total lifetime cannabis exposure (*r* = −.443, *p* = .007; Table 4), suggesting that increased lifetime cannabis use is associated with worse psychomotor function and processing speed.

## DISCUSSION

As hypothesized, cognitive performance was moderated by sex. Males, but not females, who were using cannabis at the time of study participation had worse performance on measures of intelligence, psychomotor speed, and immediate and delayed verbal recall, compared to same-sex non-using peers. Additionally, the female cannabis group performed significantly better on measures of psychomotor function and immediate verbal recall, compared to their female non-using peers. Among the observed cognitive deficits, only psychomotor function (digit symbol total score) was associated with a feature of cannabis use, i.e., total lifetime cannabis use. Significantly, the study failed to detect a main effect of cannabis use on all measures of cognition studied and thus highlighted the necessity of considering biological sex as a possible moderator of the impact of cannabis on the brain. This conclusion is further bolstered by sex differences that have been observed in neuropsychological performance more broadly (Asperholm, Högman, Rafi, & Herlitz, 2019). Notably, while differences on measures of cognition were observed, the degree of impairment did not reach a clinically impaired range, such as the level of impairment that is observed in major neurological disorders (i.e., stroke, dementia).

Causality cannot be inferred due to the cross-sectional nature of the study. Therefore, there are several possible explanations for the study findings. First, individual differences in cognitive performance may increase the likelihood of the initiation of cannabis use. This interpretation is supported in that the features of cannabis use, such as age of cannabis initiation and time since last use, were limited in their association with observed decreases in cognitive performance.

A second explanation is that cannabis use causes decreased cognitive performance in males and that women are less sensitive to the cognitive effects of cannabis. For example, while the female groups failed to differ on intellectual function, the male cannabis group was observed to have a 10-point deficit compared to the male healthy control group. These findings align with work by Lisdahl and Prince (2012), who observed neurocognitive deficits more pronounced in males, compared to females, after a week of abstinence from cannabis (Lisdahl & Price, 2012). Also, findings align with longitudinal investigations by Meier et al. in which decreases in full-scale IQ were observed in cannabis users, both before and after controlling for years of education (Meier et al., 2012). Findings also converge with those from experimental designs, collapsing across sex, involving the acute administration of cannabinoids in which acute THC administration caused significantly decreased immediate verbal recall (D’Souza et al., 2004).

THC is generally lipophilic and stored in fat cells (Huestis, 2007; Kreuz & Axelrod, 1973). Since females generally have greater body fat compared to men (Votruba & Jensen, 2007), more cannabis may be retained by fat cells of the body instead of penetrating the brain, as suggested previously by Davis and Fattore (Davis & Fattore, 2015). Additionally, it is possible that metabolism of cannabis metabolites may differ in males and females such that males are more susceptible to some of the effects of cannabis (Fattore & Fratta, 2010). Supporting this view is the finding that the male and female cannabis groups did not differ on features of cannabis use, such as time since last use, use over the past month or time since initiation. Further, no significant differences were observed between the female cannabis and female healthy control groups on measures of intelligence, delayed recall, retention, and discrimination recognition. Notably, compared to non-using peers, the female cannabis use group had significantly better performance on measures of psychomotor speed and immediate verbal recall with medium effect sizes. It is important to acknowledge that stage of menstrual cycle was not assessed in this study. Therefore, it is possible that differences in menstrual cycle influences cognitive performance as has been observed in non-cannabis using females (Souza, Ramos, Hara, Stumpf, & Rocha, 2012). Additionally, cannabis appears to alter the regulation of the hypothalamic-pituitary-ovarian axis which could impact the endocannabinoid system and cognitive performance (Brents, 2016; Gorzalka & Dang, 2012). Future research is needed to clarify the relationship between menstrual cycle, cognition, and cannabis use.

There were two unexpected findings. First, a main effect of group was not observed on any of the included cognitive measures. It is possible that collapsing across biological sex causes a washout of any potential deficits, as evident by the group by biological sex interaction. This, along with the underrepresentation of female participants in research, may also be responsible for the inconsistent observations in the literature regarding the impact of regular cannabis use on cognition.

A second unexpected finding was that the female cannabis group performed significantly better on measures of psychomotor function and immediate verbal recall (total immediate recall) compared to their female non-using peers. In a previous study, which included a subset of samples in this analysis, females were observed to have greater levels of total N-acetylaspartate (NAA), a marker of functioning neurons, although the interaction between cannabis use and sex did not reach significance (Newman et al., 2019). Additionally, while a different domain of working memory, previous work by Makela and colleagues (2006) demonstrated similarly increased performance on spatial working memory in women, but not men, with the acute administration of THC (Makela et al., 2006). Also, in both females and males, Crane et al. observed earlier age of cannabis onset was associated with better decision-making (Crane et al., 2015).

There are several important limitations to acknowledge about this study. Given the cross-sectional nature of the study, a causal link between the observed cognitive deficits and cannabis use could not be determined. Future research would benefit from longitudinal and twin-study designs with larger sample sizes. This study also did not evaluate the influence of psychiatric comorbidity or subthreshold symptoms for mental health disorders. Therefore, findings may not generalize to samples with older long-term users or users with comorbid and/or subthreshold psychiatric disorders. Population samples indicate very high lifetime rates of psychiatric comorbidity, approaching 90%, in persons with cannabis dependence (Agosti, Nunes, & Levin, 2002), suggesting the necessity for future research to investigate the relationship between psychiatric comorbidity and cognition. This study also evaluated past week alcohol use and the average number of cigarettes consumed per day at the time of study participation. It would be important for future research to explore longer periods of time prior to study participation in regard to the influence of cigarette and alcohol use. This study attempted to evaluate the extent study participants were exposed to high THC content material through the inclusion of “wax”. Future research would be enhanced by the identification of biomarkers sensitive to remote use, and types of cannabis used by participants. Future research would also benefit from using updated measures, such as the DSM-5. This study also had a high heterogeneity of frequency of cannabis use in both of the cannabis use groups. It is possible that frequent cannabis use is associated with blunted neurocognitive effects compared to infrequent users (D’Souza et al., 2008). Thus, future studies should more systematically investigate the interaction between biological sex and disordered versus infrequent use. Finally, this study was powered to detect only moderate to large effect sizes, and therefore it is imperative that future research build on these findings with larger sample sizes.

Despite these limitations, the study findings suggest that there are sex-dependent cognitive impairments associated with cannabis use. Specifically, worse cognitive performance was most robustly observed in the male cannabis use group on measures of intellectual function, psychomotor speed, and verbal learning and memory. Further, in light of the increasing rates of cannabis use and changes in public policy, these findings highlight the important complexities in the relationship between cannabis and cognition.

## Figures and Tables

**Table 1.** Demographic information

	HC (*n* = 40)	CB (*n* = 40)	*P*/Chi-square

**Demographic information**			
Age (mean ± SD)	22.7 ± 4.4	21.1 ± 3.7	.07
Sex (Male:Female)	17:23	18:22	.82
Race*	25:4:7:0:1:2:1	23:7:7:1:0:2:0	.69
Ethnicity (H:NH:UK)	6:34:0	2:37:1	.15
Education (mean ± SD)	15.5 ± 2.4	14.0 ± 1.9	**.003** *
BMI (mean ± SD)	24.9 ± 5.3	23.7 ± 4.5	.30
**Cannabis use features**			
Age (years) of initiation	19.0 ± 1.8 (Range: 17–22)	16.1 ± 2.2 (Range: 9–21)	**.001** *
Recent use (past month no. of occasions of use)	0.0 ± 0.0	31.1 ± 24.8	**<.001** *
Total lifetime CB exposure	5.2 ± 5.1 (Range: 1–15)	1149.3 ± 1762.5 (Range: 60–10000)	**<.001** *
Current CB abuse/dependence per DSM-IV	0.0 ± 0.0	23	**<.001** *
Days since last cannabis use	1213.2 ± 2158.7 (Range: 109–6906)	1.7 ± 1.6 (Range: 0–8)	.13
Positive urine toxicology for cannabinoids	0	36	**<.001** *
Ever used wax (0 = no; 1 = yes)	0.0 ± 0.0	9.8 ± .4	**<.001** *
Recent wax use (past 6 months)	0.0 ± 0.0	7.8 ± 15.0	**.003** *
**Days since**			
**Alcohol**	160.9 ± 554.7 (38 endorsed use)	20.2 ± 54.0	.13
**Tobacco**	1211.4 ± 1714.8 (12 endorsed use)	159.8 ± 427.4 (20 endorsed use)	.06
**Caffeine**	84.3 ± 335.1	6.0 ± 24.8 (37 endorsed use)	.15

*SD* = standard deviation.

* Race breakdown: Caucasian: Black/African American: Asian: American Indian/Alaska Native: Native Hawaiian or Other Pacific Islander: More than one race: Unknown; H = Hispanic; NH = not Hispanic; UK = unknown.

**Table 2.** Demographic features: male and female cannabis use groups

	Male CB group (*n* = 18; mean ± SD)	Female CB group (*n* = 22; mean ± SD)	*P*

**Demographic information**			
Age (years; mean ± SD)	21.3 ± 4.6	20.8 ± 2.8	.67
Education (years; mean ± SD)	13.8 ± 1.6	14.1 ± 2.1	.56
BMI (mean ± SD)	23.8 ± 3.2	23.7 ± 5.4	.93
**Cannabis use features**			
Age (years) of initiation	16.1 ± 1.8 (Range: 13–20)	16.0 ± 2.6 (Range: 9–21)	.90
Recent use (past month no. of occasions of use)	34.5 ± 30.2 (Range: 6–100)	28.3 ± 19.7 (Range: 4.5–90)	.44
Total lifetime CB exposure	1356.8 ± 2340.5 (Range: 60–10000)	979.6 ± 1126.1 (Range: 150–4200)	.51
Current CB abuse/dependence per DSM-IV	9	14	.39
Positive urine toxicology for cannabinoids	17	19	.08
Days since last CB use	1.2 ± 1.0 (Range: 0–4)	2.0 ± 1.9 (Range: 0–8)	.09
Ever used wax (0 = no; 1 = yes)	.9 ± .3	.7 ± .5	.13
Recent wax use (past 6 months)	7.6 ± 11.3 (Range: 0–45)	7.9 ± 17.7 (Range: 0–80)	.95
**Days since**			
Alcohol	14.7 ± 42.8	24.6 ± 62.0	.58
Tobacco	87.5 ± 216.4 (11 endorsed use)	248.1 ± 599.5 (9 endorsed use)	.42
Caffeine	3.3 ± 7.3 (16 endorsed use)	8.2 ± 32.5	.56

CB = cannabis; SD = standard deviation.

* *p* < .05.

**Table 3.** Cognitive measures by group

	Groups			Males			Females		
Cognitive measure	HC	CB	*P*	HC	CB	*P*	HC	CB	*P*

**WASI IQ**	114.7 ± 10.3	109.9 ± 8.9	.06	118.6 ± 7.5	108.2 ± 9.8	<.001	111.9 ± 11.34	111.2 ± 8.2	.80
**Digit symbol**	90.8 ± 14.7	89.2 ± 14.4	.38	92.5 ± 16.2	81.6 ± 16.2	<.001	89.4 ± 13.6	95.3 ± 9.7	<.001
**Digit Span**	20.3 ± 4.0	19.8 ± 3.8	.60	19.8 ± 3.3	18.2 ± 3.1	ns	20.7 ± 4.5	21.1 ± 3.8	ns
**HVLT recall**									
**Immediate**	28.5 ± 3.4	27.7 ± 3.9	.58	28.2 ± 2.3	25.8 ± 3.9	<.001	28.7 ± 4.1	29.3 ± 4.1	.02
**Delayed**	10.8 ± 1.5	10.2 ± 1.5	.22	10.9 ± 1.1	9.4 ± 1.8	<.001	10.7 ± 1.8	10.8 ± 0.9	.94
**Cued recognition recall**	11.7 ± .7	11.6 ± .8	.85	11.5 ± .8	11.3 ± 1.0	ns	11.7 ± .5	11.9 ± .4	ns
**Retention percent T score**	51.5 ± 6.5	48.6 ± 11.5	.25	53.2 ± 5.5	45.3 ± 12.7	<.001	50.3 ± 7.1	51.2 ± 9.9	.14
**Discrimination index T score**	53.0 ± 7.6	51.0 ± 9.2	.76	51.4 ± 9.8	46.2 ± 10.7	ns	54.2 ± 5.4	54.8 ± 5.4	ns

HC = healthy control group; CB = cannabis use group.

*Note: p* = significance value from post-hoc pairwise comparisons in cases with significant interactions between group and biological sex; *p* = ns is indicative of domains in which post-hoc testing was not completed due to non-significant group × biological sex interactions; 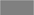 dark gray shading indicates when the cannabis group performed below the same-sex control group; 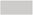 the light gray shading indicates when the cannabis group performed better than the same-sex control group. All scores are the total/raw scores except for the WASI IQ and where indicated as a T-Score (i.e., retention percent).

**Table 4.** Correlations between cognitive measures with observed group differences and features of cannabis use: combined cannabis groups

Both Cannabis groups combined: r (p)						
	Features of Cannabis use					
Cognitive measure	Total lifetime exposure	Time since last use (days)	Past month use	Age of cannabis initiation	Tried wax (0 = no, 1 = yes)	Wax uses over past 6 months

WASI IQ	−.008 (.961)	.177 (.288)	.070 (.675)	.027 (.870)	−.170 (.309)	.046 (.784)
Digit Symbol	**−.433 (.007)**	.268 (.104)	−.183 (.271)	−.183 (.272)	−.105 (.529)	.139 (.405)
HVLT: Delayed Recall	.086 (.608)	−.010 (.951)	−.013 (.940)	.093 (.577)	−.097 (.563)	.088 (.600)
HVLT: Retention	.143 (.391)	−.009 (.958)	.033 (.845)	.108 (.520)	.011 (.947)	.001 (.994)

WASI IQ = measure of intellectual function; HVLT = Hopkins Verbal Learning Test.

*Note:* Bold indicates significant *p* value (*p* < .05).
